# Novel Gene Expression Profile of Women with Intrinsic Skin Youthfulness by Whole Transcriptome Sequencing

**DOI:** 10.1371/journal.pone.0165913

**Published:** 2016-11-09

**Authors:** Jin Xu, Robert C. Spitale, Linna Guan, Ryan A. Flynn, Eduardo A. Torre, Rui Li, Inbar Raber, Kun Qu, Dale Kern, Helen E. Knaggs, Howard Y. Chang, Anne Lynn S. Chang

**Affiliations:** 1 Department of Dermatology, Stanford University School of Medicine, Redwood City, California, United States of America; 2 Department of Pharmaceutical Sciences, University of California Irvine, Irvine, California, United States of America; 3 Nu Skin International, Provo, Utah, United States of America; University of Colorado Denver School of Medicine, UNITED STATES

## Abstract

While much is known about genes that promote aging, little is known about genes that protect against or prevent aging, particularly in human skin. The main objective of this study was to perform an unbiased, whole transcriptome search for genes that associate with intrinsic skin youthfulness. To accomplish this, healthy women (n = 122) of European descent, ages 18–89 years with Fitzpatrick skin type I/II were examined for facial skin aging parameters and clinical covariates, including smoking and ultraviolet exposure. Skin youthfulness was defined as the top 10% of individuals whose assessed skin aging features were most discrepant with their chronological ages. Skin biopsies from sun-protected inner arm were subjected to 3’-end sequencing for expression quantification, with results verified by quantitative reverse transcriptase-polymerase chain reaction. Unbiased clustering revealed gene expression signatures characteristic of older women with skin youthfulness (n = 12) compared to older women without skin youthfulness (n = 33), after accounting for gene expression changes associated with chronological age alone. Gene set analysis was performed using Genomica open-access software. This study identified a novel set of candidate skin youthfulness genes demonstrating differences between SY and non-SY group, including pleckstrin homology like domain family A member 1 (PHLDA1) (p = 2.4x10^-5^), a follicle stem cell marker, and hyaluronan synthase 2-anti-sense 1 (HAS2-AS1) (p = 0.00105), a non-coding RNA that is part of the hyaluronan synthesis pathway. We show that immunologic gene sets are the most significantly altered in skin youthfulness (with the most significant gene set p = 2.4x10^-5^), suggesting the immune system plays an important role in skin youthfulness, a finding that has not previously been recognized. These results are a valuable resource from which multiple future studies may be undertaken to better understand the mechanisms that promote skin youthfulness in humans.

## Introduction

While many genetic mechanisms promoting aging are known, the pathways protecting against aging in humans are not well understood. A few studies have shown that metabolism and lipid related genes associate with human longevity [1,2]. The skin is an ideal model to study aging-protective pathways due to the ease of clinical inspection and biopsy compared to other organ systems. Recently, a genome-wide association study in an Ashkenazi population of centenarians identified single nucleotide polymorphisms associated (SNPs) with skin youthfulness (SY) including a SNP in the intronic region of a voltage-gated potassium channel [3].

A number of genes have been associated with protection against aging in animal models. In mice, suppression of the NFkB pathway can rejuvenate skin [4], an effect partially mimicked by broadband light treatments in human skin [5]. Another animal model for aging, the naked mole rat, possesses exceptional longevity and resistance to cancer, an age-associated condition, thought to be due to high expression of ultra-high molecular weight hyaluronan [6]. However, data on the mechanisms promoting SY is scant.

The main objective of this study was to identify intrinsic gene pathways that may protect against human skin aging. We examined gene expression profiles by 3’-end whole transcriptome sequencing of sun-protected skin, comparing profiles of older individuals with and without clinically visible SY phenotype.

## Materials and Methods

### Human subjects and sample collection

Stanford Human Subjects Panel approval (IRB #17885, IRB Panel: IRB-1) and written informed consent was obtained from all participants prior to study related procedures. This study was conducted in accordance with the Declaration of Helsinki Principles. Healthy female volunteers of European descent (defined as possessing 4 grandparents from Europe) and Fitzpatrick skin type I/II ranging from age 18 to 89 years were enrolled (n = 122). Exclusion criteria included individuals with topical anti-aging usage, including tretinoin and retinol, and history of cosmetic procedures (such as laser resurfacing, chemical peels, facelift). Covariates for skin aging were recorded, including estimates of cumulative sun exposure, personal history of skin cancer, body mass index, and smoking history. Clinical histories were recorded in Microsoft Excel, version 15.0, Redmond, WA). The individuals whose photographs are presented in this manuscript have given written informed consent to publish these case details.

Inclusion criteria for enrollment included: Fitzpatrick skin type I/II, female, non-obese body mass index. Exclusion criteria included: uncontrolled medical problems, history of cosmetic procedures to face (includes but not limited to laser, peels and surgeries), history of anti-aging or bleaching topical product within 2 weeks of enrollment, use of hormonal therapy within 2 weeks of enrollment, use of retinoid within 1 month of enrollment, pregnant or lactating. After eligibility was verified, all enrollees were photographed by Canfield Visia Imaging System with Complexion Analysis (Fairfield, NJ) using the same settings for all participants.

Skin biopsies by punch technique were performed after infiltration of the subcutaneous skin with 1% lidocaine with epinephrine diluted 1:1,000,000 for local anesthesia. The skin biopsies were 4 mm in diameter and included both dermis and epidermis. The samples were bisected, and half was preserved in formalin solution for staining with hematoxylin and eosin while the other half was preserved in RNAlater (Ambion, catalog number AM7022, Grand Island, NY). Epidermal thickness was assessed using light microscopy at 200x magnification. Skin autofluorescence for advanced glycation end products was performed on clean volar forearm skin using the AGE Reader (DiagnOptics Technologies, Groningen, The Netherlands).

**Fig 1:**
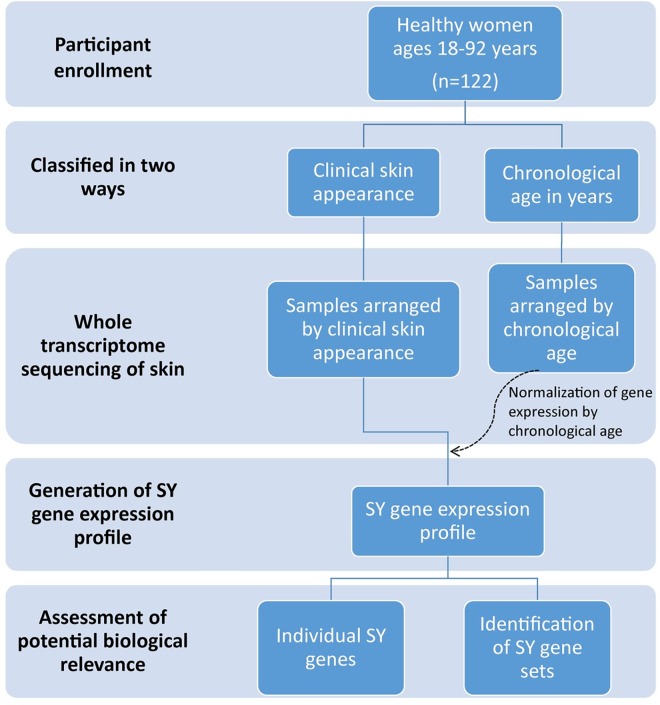
Summary of the study flow. This figure depicts the overall design of the study.

The overall study flow is shown in Fig 1.

### Skin age assessments

**Fig 2:**
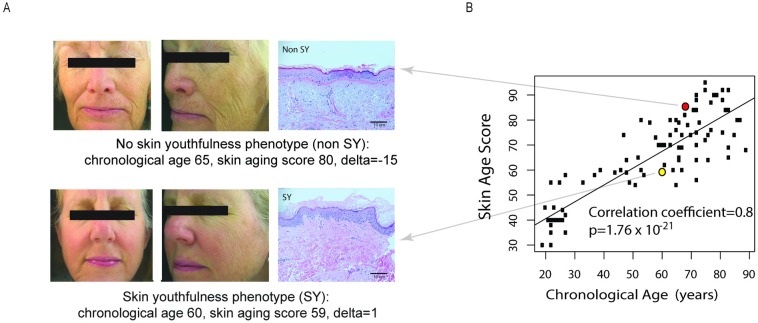
Determination of the skin youthfulness (SY) phenotype. (A) Example of facial photographs and skin histology from individuals with youthfulness (SY) and without SY (non-SY) individuals. Upper panels show an example of individual without SY (chronological age (CA) 65 years, assessed skin age score (SAS) 80, delta (CA minus SAS) = -15, residual of delta (r_delta_) = -8; lower panel shows an example of an individual with SY, CA = 60 years, SAS 59, delta (CA minus SAS) = 1, r_delta_ = 10. Examples of skin histology, demonstrating slightly more epidermal cell layers and elastosis in SY than non-SY skin (hematoxylin and eosin stain, 200x magnification). (B) Correlation between skin age score versus chronological age. The non-SY and SY subjects shown in the panels in A are represented in the corresponding red and yellow dots respectively in Fig 2B.

To identify individuals with SY, high quality digital photographs of clean facial skin using the Canfield medical imaging software (described above) were examined by four dermatologist raters blinded to the chronological age of the volunteers (examples shown in Fig 2A). The scale used in skin aging assessments was previously shown to be reliable [3] and incorporates fine and coarse wrinkling as well as sagging. These characteristics capture both intrinsic and extrinsic skin aging parameters [7], as the genetics can influence response to environmental factors [3]. Quantitation of facial wrinkles was performed by Canfield Medical Imaging Software (VISIA Complexion Analysis) and independently confirmed by clinician-assessed skin aging scores. A global skin age score (SAS) was defined for each participant, based on the median scores of the dermatologists’ ratings.

The difference between chronological age and SAS, defined as “delta”, was used to describe the relative skin appearance to chronological age for each individual, as previously reported [3]. Since the spread of “delta” increased with chronological age, linear regression of delta was performed to assess the rate of increase of delta due to age (S1A Fig). Subsequently, the residual of the linear regression of delta (S1B Fig) on chronological age was used as the quantitative skin appearance in order to exclude the bias of chronological age [8]. Individuals with chronological age less than 30 years old served as the positive control group, termed the “young” group. For the purposes of this study, individuals with a residual of delta in the top 10% of all individuals over the age of 50 years were defined as having “SY” phenotype. The remaining individuals with a residual of delta in the lower 50% of individuals were defined as “non-SY.”

### Covariate analysis

Potential covariates of age, smoking history, ultraviolet radiation exposure [9], body mass index, and personal history of skin cancer (as this has been shown to alter sun protective behaviors [10]) were assayed for these three groups and tested by T-test or Fisher’s exact test in R. The scores for the young, SY and non-SY groups were tested by T-test in R. Results are shown in Table 1.

**Table 1:** Demographics of participants enrolled comparing SY and non-SY groups with regard to potential confounders of skin aging.

Parameter	SY group (n = 12)	Non-SY group (n = 33)	P
**Chronological age, years (SD)**	71(11)	69(9)	0.75
**Mean body mass index kg/m^2^ (SD)**	25 (3.3)	25(4)	0.71
**Smoking history: Yes (%)**	3 (25%)	13 (39%)	0.74
**Previous Skin cancer: Yes**	3 (25%)	10 (30%)	1.00
Mean lifetime UV hours accounting for UV Index*, score (SD)	36 (27)	49 (44)	0.26

There were no significant changes between SY and non-SY group in chronological age, body mass index, smoking history, previous skin cancer, or estimated lifetime ultraviolet exposure.

*Calculated by questionnaire on lifetime ultraviolet exposure using validated items [9].

### RNA extraction and 3’-seq

Total RNA from full thickness skin biopsies of the sun protected upper inner arm skin was extracted using RNAeasy Fibrous Tissue Mini Kit (Qiagen, Germantown, MD). The 3′-end sequencing for expression quantification (3’-end seq) was performed as described in [11]. In brief, mRNA was first enriched from total RNA by poly-A selection to remove the ribosomal RNA and other non-poly-A RNA. The mRNA was then fragmented to 100–200 bases. The oligo-dT-directed reverse transcription generated complementary DNAs corresponding to 3’end of polyA transcripts; the complementary DNA were amplified by PCR and subjected to deep sequencing on the Illumina Hiseq2500/200 (San Diego, CA) platform with raw reads length of 50 base pairs. Results were normalized and batch effects were removed by Combat.

### RT-qPCR

To confirm the results of the RNA sequencing, selected genes underwent RT-qPCR analysis. Total-RNA was extracted with TRIzol (Invitrogen, Grand Island, NY) followed by RNeasy column purification (Qiagen) and DNase Turbo Treatment (Ambion). RT-PCR was performed using total RNA (10ng), Taqman One Step RT-PCR master mix. Taqman assays of detected genes were as follows: ACTβ gene, Taqman Assay ID Hs99999903_m1; HAS2-AS1, Taqman Assay ID Hs03309447_m1; HAS2, Taqman Assay ID Hs00193425_m1. Reactions were performed in duplicates for each sample and data were normalized to ACTβ levels.

### Bioinformatics analysis

The raw reads from 3’-sequencing were aligned to human genome reference (hg19) using Bowtie 2 (version 2.1.0) [12] with default parameters (-D 15 -R 2 -N 0 -L 22 -i S,1,1,15). Each sample yielded 14–44 million mapped reads. Mapped reads falling in each gene region annotated by Reference Sequence (RefSeq; http://hgdownload.soe.ucsc.edu/goldenPath/hg19/database/refgene.txt.gz) were calculated using DEGseq as gene expression levels [13]. The raw count for each gene was further normalized into 10 million mappable reads. 17,321 genes with mean expression value more than 2 were kept as expressed genes and used for the skin aging expression analysis. To remove batch effects, ComBat in SVA package was applied to the gene expression matrix after log_2_ transformed [14]. (http://www.bioconductor.org/packages/release/bioc/vignettes/sva/inst/doc/sva.pdf).

To generate gene expression profiles for all individuals by chronological age (from age 18–89), age related genes were detected using limma package (R) with empirical bayes statistics for differential expression (eBayes) function, which regressed expression of each gene on chronological age [15].

To identify gene expression profiles associated with skin youthfulness, the gene expression for profiles of the SY group were compared to the non-SY group. For SY gene detection in all individuals, a linear regression of the expression measurements on chronological age was performed. The residuals from this regression were used to calculate relative gene expression. Skin youthful genes were identified using limma (R) by regression relative expression on SY phenotype (as defined above).

Unsupervised hierarchical clustering of different expression genes was performed using Cluster and visualized by TreeView. The GO terms annotation were generated using Database for Annotation, Visualization and Integrated Discovery (DAVID) Bioinformatics Resources 6.7 (http://david.abcc.ncicrf.gov/). All the correlation coefficients were calculated using Pearson’s correlation coefficient in R (version 3.0.2).

Gene set analysis was performed using Genomica open access software available at http://genomica.weizmann.ac.il/. Genomica is an analysis and visualization tool for genomic data, which can check for enrichment of a collection of gene sets that represent biological processes. Currently, there are 7 gene sets available, and one such sets is the Gene Ontology (GO) database. To apply Genomica, gene expression level from 3-seq for SY and non-SY group were first normalized and formatted into the expression matrix as required by Genomica. After loading the expression matrix, Gene Ontology annotation for each gene were loaded as gene sets. Group information were represented by “0” or “1” and loaded as experiment sets. We set the minimum expression change to 0.3 and multiple hypotheses correction to 0.1 for expression difference, no correction for experiment test. All the other parameters were left as default. The gene sets identified as significantly different between SY and SY groups were export from Genomica and visualized by Treeview as a heat map.

Functional gene groups were downloaded from Molecular Signatures Database (MSigDB) from multiple gene sets (collections 1–7 (C1-C7)) (S4 Table) consisting of well-defined and well-characterized gene sets: H aka C1: Hallmark gene sets, C2: CP (Canonical Pathways), C3: motif gene sets, C5: Gene Ontology (GO) gene sets, C7: immunological signature gene sets. C6: data mining of cancer-oriented microarray data were excluded as our study does not directly pertain to cancer.

### Immunofluorescence

To examine the protein expression of the two most interesting genes associated with SY phenotype, PHLDA1 and HAS2-AS1, formalin fixed paraffin-embedded skin samples were immunohistochemically stained with HAS2 monoclonal antibody (LifeSpan Biosciences, Inc., clone 4E7 (1:500 dilution) made in mouse) and PHLDA1 monoclonal antibody (LifeSpan Biosciences, Inc., clone EPR6674 (1:250 dilution) made in rabbit). Donkey anti-mouse IgG or donkey anti-rabbit IgG (Life Technologies AlexFluor 488) (1:100 dilution) secondary antibodies were used. The tissue was visualized with confocal microscopy on a Leica SP8 WLL Laser Scanning Microscope with an HCX PL APO **_**40 oil immersion objective. Images were arranged with ImageJ (Solms, Germany), Adobe Photoshop (San Jose, CA), and Adobe Illustrator (San Jose, CA).

### Knockdown of HAS2-AS1

To explore the effects of a novel SY candidate gene HAS2-AS1, knockdown experiments of the transcript were performed *in vitro*. Reactions of 200,000 dermal fibroblasts were nucleofected using the Amaxa Human dermal fibroblast reagent (Lonza) with 60 pmol of control or HAS2-AS1 targeting antisense oligonucleotides. Cells were incubated under normal culture conditions for 24, 48, or 72 hours after which RNA was isolated as described previously for gene expression analysis. Oligos and probe sequences (5’-3’) were as follows: HA2-AS1_1 agacaaagtatccagaaca, HAS2-AS1_2 tagatgtgacuuagcaata, HAS2-AS1_3 gtaataggacacaggtctt

## Results

### Clinical characteristics of study population

To explore whether SY group and non-SY groups were confounded by factors known to influence skin aging, factors such as chronological age, body mass index, body mass index, smoking history, previous skin cancer history, and mean lifetime ultraviolet exposure were compared between the two groups. No statistical difference was found between the two groups when comparing these factors (all p values >0.05 (Table 1).

To ensure that the skin aging assessments by the dermatologists are consistent with other methods of quantitating skin aging, the SY and non-SY groups as determined by dermatologists were examined for wrinkle score by Visia computer software analysis and mean epidermal thickness on histology, because skin aging is associated with increased wrinkling and decreased epidermal thickness [4]. The mean skin age scores for those with and without SY were significantly different, at 61 years (standard deviation 5) and 81 years (standard deviation 8) respectively, p = 2.335x10^-11^. The mean wrinkle score by Canfield VISIA Complexion Analysis computer software between SY and non-SY was significantly different between SY and non-SY groups, at 93^rd^ percentile (standard deviation 10.6) and 73^rd^ percentile (standard deviation 22), p = 0.000382, consistent with the dermatologists’ ratings. The mean (standard deviation) epidermal thickness between SY and non-SY was 12.5 (1.96) and 9.7 (2.91) cell layers respectively, p = 0.000820 (Table 2). Examples of SY and non-SY individuals, with corresponding histology, and skin age score versus chronological age, are shown in Fig 2A and 2B.

**Table 2:** Known attributes of skin aging are significantly different between skin youthfulness (SY) and non-SY group phenotype.

Parameter	SY group (n = 12)	Non-SY group (n = 33)	P
**Mean skin age score (SAS), years (SD)**	61.0 (5.0)	81.0 (8.0)	2.34x10^-11^
**Mean percentile VISIA computer software wrinkle score (SD)**	93.0 (10.6)	73.0 (22.0)	0.00038
**Mean epidermal thickness, by cell layers (SD)**	12.50 (1.96)	9.70 (2.91)	0.0082

Assessments were made by dermatologists’ clinical evaluation, computer based complexion analysis software, and histological examination.

### Establishment of a global gene expression profile based on chronological age

To assess the unique gene expression profile of SY individuals, as determined by the dermatologists’ assessments, we first needed to define the gene expression changes that occur with chronological aging, regardless of skin appearance. Only after assessing the gene expression changes that occur with chronological aging could we explore the genetic differences between the skin samples from SY individuals and the non-SY individuals.

Whole transcriptome sequencing by 3’-end sequencing, a method that allows for recovery of long non-coding RNAs as well as coding RNAs [16], was performed on full thickness sun-protected inner arm skin from all study participants. The data is accessible on Gene Expression Omnibus (GEO) under accession number GSE85861. Results on average revealed approximately 27 million mappable reads for each sample, with detection of 16,322–19,001 genes among the samples. The mean expression value was >2 in all the samples after normalization to sequencing depth.

**Fig 3:**
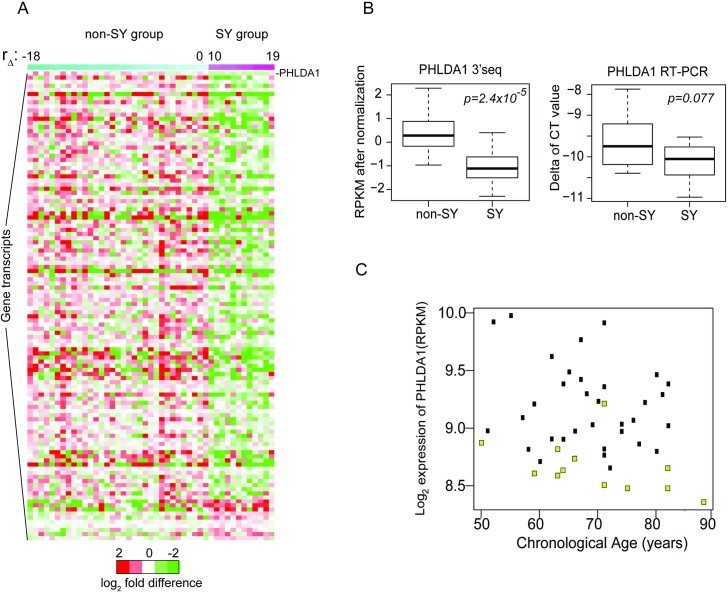
Gene expression profiles of SY (n = 12) versus non-SY individuals (n = 33). (A) Heat map of gene expression profiles showing differences between SY and non-SY group, ranked by r_delta_. Gene transcripts most significantly altered in SY are listed in descending order, with the most significant one being PHLDA1. S3A Table shows the full list of gene transcripts. (B) Differences in transcript levels of PHLDA1 between SY (n = 12) and non-SY (n = 33) groups by 3’seq and confirmed by RT-PCR. Expression levels by RPKM were normalized by batch and age effects as described in Methods section (left side of Fig 3B). For RT-PCR, the delta Ct values after normalization to internal control gene, actin-β are shown (right side of Fig 3B). (C) To examine levels of PHLDA1 in SY individuals over a range of ages, transcript levels were plotted versus chronological age (Fig 3C). SY individuals exhibited lower expression levels of PHLDA1 across multiple decades (yellow dot = SY individual, black dot = non-SY individual).

Using an false discovery rate (FDR) <0.01, 342 genes were detected which changed with chronological age, shown as a heat map in Fig 3. There were 186 genes increased and 156 decreased (S1A and S1B Table). Of these 186 genes, 177 are coding RNAs and 9 are non-coding RNAs. Notably, the heat map demonstrates that the most noticeable changes in gene expression profiles occur around 40 years of age, a finding consistent with prior human aging studies in non-skin organs [17,18].

When all the significantly increased transcripts were analyzed by gene ontology (GO) terms, the most significant terms fell under the theme of cell adhesion (p = 1.05x10^-10^), biological adhesion (p = 1.09x10^-10^), positive regulation of response to stimulus (p = 2.78x10^-9^), extracellular cellular organization (includes collagen pathways) (p = 1.3x10^-8^), positive regulation of immune response (p = 3.28x10-8), activation of immune response (p = 4.4x10-8) (S2A Table). The top nine biologic themes most significantly decreased with age involve genes related to pigmentation (S2B Table).

Each of these processes of cell adhesion, extracellular structure organization and immune response dysregulation have been reported in the literature to associate with skin aging [19,20,21], suggesting validity of our results. Dyspigmentation is clinically associated with both intrinsic and extrinsic aging of skin and can be manifest as hyperpigmentation (e.g. melanonychia, melasma or lentigos) or hypopigmentation (e.g. canities or guttate hypomelanosis) [22].

Particular transcripts whose expression levels increased with chronological age include several with known aging function. For instance, IRAK2, TGFbetaR3, EDA2R and TLR4, are positive regulators of NFkB transcription factor activity. NFkB pathway expression is known to associated with restoration of youthful skin phenotype in mice [4] and NFkB-related gene expression is associated with reversal of skin aging in human skin using broadband light [5].

### Identification of a novel global gene expression profile of intrinsic human skin youthfulness

To identify the gene expression profile associated with intrinsic skin youthfulness, the individuals in this study were ranked by skin aging phenotype, namely residual of delta (r_delta_) as shown in S1B Fig. Delta, as described in Methods section, is the difference between assessed skin age score (SAS) and chronological age (CA) (S1A Fig). Residual of delta (r_delta_) was used because the variance of delta increased with chronological age (S1B Fig). Among the participants age 50 years and older, the subjects with the top 10^th^ percentile of _rdelta_ values were defined as SY (n = 12), and those in the 50^th^ percentile or lower of r_delta_ were defined as non-SY (n = 33). Since we had previously created a reference gene expression profile sorting all study participants by chronological age, we were now able to subtract out the gene expression changes due to chronological age alone [23,24]. The remaining gene expression changes were compared between the SY and non-SY group to generate the novel gene expression profile of SY.

Overall, 114 transcripts were altered in the SY group, at a level of p<0.01, with 104 transcripts decreased, and 10 transcripts increased. Fig 3A is a heat-map incorporating the results of the gene expression analysis, listed in order of p-values of genes. The complete list of SY transcripts can be found in S3A and S3B Table. Ninety-eight transcripts were coding RNAs, 16 were non-coding RNAs. Five transcripts were pseudogenes. This set of SY genes was distinct from the chronological aging genes, with only five genes overlapping (S3 Fig). The data is accessible on the Gene Expression Omnibus (GEO), accession number is GSE85861.

### Selected genes of interest for intrinsic human skin youthfulness: PHLDA1 and HAS2-AS1

The transcript most significantly altered with SY was PHLDA1, a marker for hair follicle stem cells in the hair bulge [25,26]. Fig 3B shows verification of the 3’-seq results for the most significantly altered gene, PHLDA1 by RT-PCR. The difference in PHLDA1 transcript levels between SY and non-SY groups by 3’-seq was significant, by linear regression model, with p = 2.4x10^-5^ (Fig 3B, left side). Expression levels by RPKM were normalized by batch and age effects as described in Methods section (Fig 3B, left side). The difference in PHLDA1 transcript levels between SY and non-SY groups by RT-PCR was also significant, by Wilcoxon rank sum test, p = 0.07667 (Fig 3B, right side) after normalization to internal control gene, actin-beta.

To examine levels of PHLDA1 in SY individuals over a range of ages, transcript levels were plotted versus chronological age (Fig 3C). SY individuals exhibited lower expression levels of PHLDA1 across multiple decades compared to non-SY individuals (yellow dot = SY individual, black dot = non-SY individual). There was no significant correlation between PHLDA1 expression levels and chronological age (correlation coefficient = -0.227, p = 0.1345).

PHLDA1 plays a role in the anti-apoptotic effects of insulin-like growth factor-1, important in multiple aging functions [27]. To assess if there are differences in PHLDA1 protein levels in SY versus non-SY skin, immunofluorescence studies in formalin fixed paraffin embedded skin of SY and non-SY individuals were performed. In all skin samples tested (n = 5 SY, n = 5 non-SY), a positive signal in the nuclei of hair follicle stem cells and lower levels in the cytoplasm of keratinocytes were detected, consistent with other reports. (www.proteinatlas.org/ENSG00000139289-PHLDA1/tissue/skin). However, there were no discernable differences in levels of immunohistochemical staining between SY and non-SY (data not shown), and the exact role of PHLDA1 in the SY phenotype remains to be determined.

Of the 16 non-coding gene transcripts that were altered in SY, we examined the long non-coding RNA hyaluronan synthase2-antisense 1 (HAS2-AS1) in more depth because of the known association of hyaluronan synthase pathway with aging-related phenomena [6,28,29]. For instance, ultra high molecular weight hyaluronan is reported to contribute to the longevity of naked mole rats [30,31]. The role of HAS2-AS1 in human skin aging is less clear, although it has been reported to mediate wound healing in human skin, a process that may be impaired in older individuals [29].

**Fig 4:**
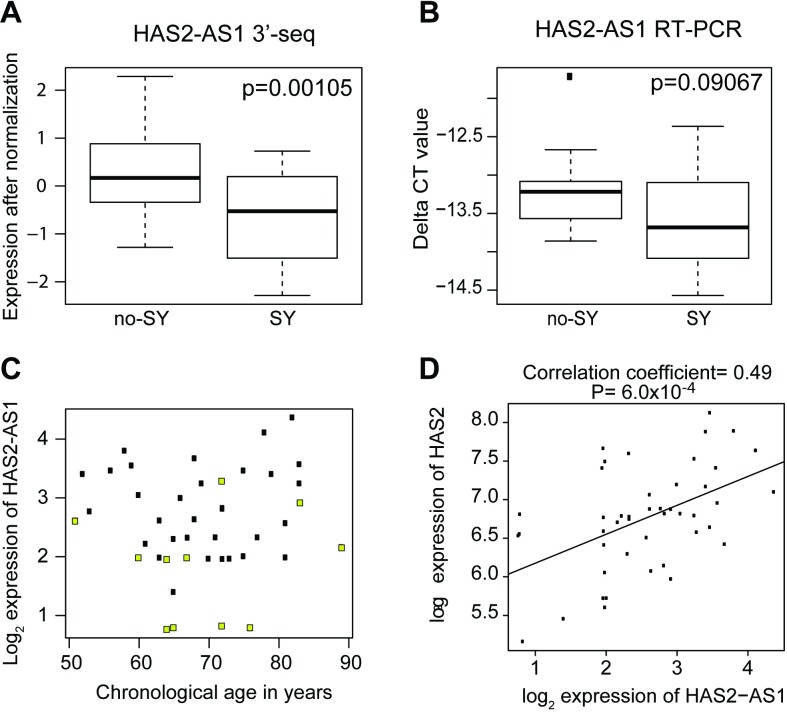
HAS2-AS1 expression is decreased in SY and increases with increasing HAS2 transcript level. (A) Expression difference of HAS2-AS1 between SY group (n = 12) and no-SY (n = 33) group by 3’seq, p = 0.00105. (B) By RT-PCR, expression of HAS2-AS1 was decreased in SY group (n = 10), with trend toward statistical significance, p = 0.09067. (C) Scatterplot of HAS2-AS1 expression in SY individuals (yellow dots) shows this decreased expression of HAS2-AS1 occurs across different chronological ages. (d) In general, there is a positive correlation between HAS2-AS1 and HAS2 levels among all patients (n = 45), correlation coefficient (R) = 0.49, p = 0.00063.

HAS2-AS1 is expressed in human keratinocytes [32] and cultured fibroblasts (GTEX portal (accessed on May 5, 2016)) and therefore may play a role in regulating hyaluronan. Clinically, hyaluronic acid is commonly injected as a filler to reduce the appearance of facial wrinkles in patients, a phenotype associated with aging [28,33]. As a first step, we verified that HAS2-AS1 levels were indeed lower in SY individuals compared to non-SY group by RT-PCR (Fig 4). While the p-values were not significantly different between the SY and non-SY group, the directionality of the transcript levels were the same by both 3’-seq and RT-PCR. This lack of significance in HAS1-AS2 levels could be due to the fact that long non-coding RNAs are often expressed at extremely low copy number per cell (including <1 transcript per cell), consistent with their roles in regulating genes [34,35].

The lowered HAS2-AS1 levels occurred in SY individuals of different chronological ages (Fig 4C). Since HAS2-AS1 pairs with HAS2, we examined the correlation of HAS2-AS1 levels with HAS2 levels and found that they were positively correlated, with correlation coefficient = 0.49, p = 6.0x10^-4^ (Fig 4D). To explore the potential effects of HAS2-AS1 in human fibroblasts, knockdown of HAS2-AS1 using anti-sense oligonucleotides was performed in cultured dermal fibroblasts. However, these studies showed non-significant decreases in HAS2 transcript levels by RT-PCR after 24, 48 or 72 hours of treatment with HAS2-AS1 oligonucleotides and with two biological replicates due to the small knockdown efficiency of only 15%. We also examined effects of the knockdown on the putative target of HAS2-AS1, specifically HAS2, but there was no significant reduction, thereby precluding any definitive conclusions. Nevertheless, this data does not exclude the possibility that the HAS2 and HAS2-AS1 gene pair may have regulatory function in human skin [36].

Additional individual SY genes with interesting themes or function are displayed in Table 3. These include genes whose expressions are altered in the broadband light treatment, a treatment used in the clinical setting to rejuvenate skin, along with genes that participate in pathways that have known aging functions such as NFkB pathway [4].

**Table 3:** Additional individual SY genes with interesting themes or function.

Aging related pathway	Selected references	Number of genes	SY Genes (bold font indicates increased levels in SY, non-bold font indicates decreased transcript levels)	
Glycoprotein biosynthetic process or glycoprotein metabolic process	Huang *et al*., 2009, accessed on DAVID Bioinformatics Resources 6.7	158 and202 respectively (GO)	• PHLDA1 • NDST2 • ST6GAL1 • DPM3 • GCNT1	
Hyaluronan synthesis pathway	Gorbunova *et al*., 2014	N/A	**HAS2-AS1**	
NFkB targets	Adler *et al*., 2007	610 (MSigDB)	• NTRK3 • PCDH10 • OGG1	
Broadband light related rejuvenation genes	Chang *et al*., 2013	3,531 (Chang *et al*., 2013)	• CFB • LOC283335 • SH3BP4 • MRPS12 • ACVRL1 • PIWIL4 • DPM3 • NEK1 • ECE2 • EML3 • BAMBI • FAM189A2	• DGKD • SOD2 • STXBP1 • LAGE3 • NTRK3 • CCDC88A • RASSF4 • PLEKHJ1 • PADI2 • **UTP11L** • **BAG5** • **CDT1**

**Fig 5:**
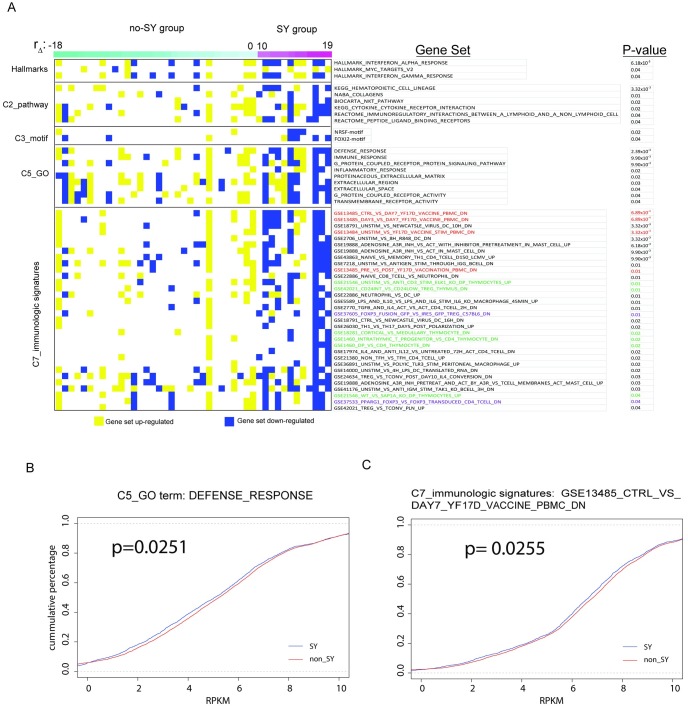
Gene sets that are the most significantly different between SY and non-SY are immune related. (A) Results of gene set analysis ranked by p-value. (B) Cumulative plot of gene expression levels for genes within the GO term “DEFENSE_RESPONSE”. (D) Cumulative plot of gene expression for genes within the set GSE13485_CTRL_VS_DAY7_YF17D_VACCINE_PBMC_DN from C7: immunologic signatures collection. In both (C) and (D), SY patients have globally decreased levels of gene expression from these two gene sets compared to non-SY group.

SY transcripts with known association to aging function, out of the 114 SY transcripts identified by 3’-seq that are not included in the Gene Sets depicted in Fig 5.

### Gene set analysis for intrinsic skin youthfulness demonstrates significance of immunological gene sets

To further examine the results of the whole transcriptome profiles of SY individuals, we searched for clusters of SY genes grouped by functional features using gene sets from Molecular Signatures Database (MSigDB). The full list of gene sets analyzed is shown in S4 Table and comprised the following collections: hallmark gene sets (H, consisting of 50 gene sets), canonical pathways (C2, consisting of 1,330 gene sets), motif gene sets (C3, 836 gene sets), Gene Ontology gene sets (C5, 1,454 gene sets) and immunologic signatures gene sets (C7, 4,872 gene sets). The most significantly altered gene sets ranked by p-values are shown in Fig 5. A full list of significant gene sets is shown in S4 Table.

Gene set analysis revealed that the most significantly altered gene sets are related to immunologic signatures found in C7. Specifically, the most significantly altered gene set was “GSE13485_CTRL_VS_DAY7_YF17D_VACCINE_PBMC_DN” (p = 6.89x10^-4^) (Fig 5A). This set is derived from pre- versus post-vaccination peripheral blood mononuclear cells (PBMCs) of human volunteers (http://www.ncbi.nlm.nih.gov/geo/query/acc.cgi?acc=GSE13485, accessed July 1, 2016). Additional vaccine related gene sets that were statistically significant are shown with red font in Fig 5A.

Six gene sets that were significantly altered in SY under the immunological signatures gene set (C7) pertained to thymocyte stimulation and are shown in green font in Fig 5A. Two gene sets in the immunological signatures gene set (C7) that were significantly changed in SY were related FOXP3, a master regulator in the development and function of regulatory T cells, and are shown in purple font (Fig 5A).

Within the Gene Ontology (GO) category (C5), the most significant terms were related to defense response (p = 2.39x10^-3^) and immune response (9.90x10^-3^) (Fig 5A), consistent with the significant gene sets identified through C7. The cumulative plots shown in Fig 5B and 5C are examples of the two GO terms, Defense Response and Immune Response and demonstrates that the expression of genes in these two gene sets are globally and significantly different in SY individuals (p = 0.0251 for Defense Response, and p = 0.0255 for Immune response).

## Discussion and Conclusions

The gene expression profiles of SY reported here represent a springboard for multiple future lines of investigation to better understand how to promote skin youthfulness. Future studies that utilize larger numbers of participants would increase statistical power to detect differences in SY versus non-SY individuals.

Caveats to this current study include the fact that the skin samples were not separated into dermis or epidermis, and doing so may enable the determination of relative contributions of the cells in each layer to the SY phenotype. In addition, although this is a relatively large human study, the effects of SY related genes are small compared to genes associated with chronological age. Therefore, a larger sample size would provide greater power to determine SY related genes.

HAS2-AS1 knockdown experiments were not successful possibly due to the location of HAS2-AS1 in the nucleus and/or its low copy number per cell [37]. Nevertheless, HAS2-AS1 is a fascinating transcript worthy of future study. Published data has suggested that HAS2-AS1 may induce HAS2 expression [6] but how this occurs is still unclear. The HAS2-AS1 transcribed region overlaps a CCCTC-binding factor (CTCF) motif, the binding site for an 11-zinc finger domain DNA-binding protein that has importance in regulating chromosome looping and gene regulation, including activation, repression, and insulation of genes such as the H19/Igf2 locus [38]. CTCF can also interact with promoters such as MYC promoter[39]. In addition, more recent work has shown that CTCF is a downstream component of the NFkB pathway and involved in cell fate determination [40]. Additional studies in the future may shed light on the intriguing connection between HAS2-AS1 and skin youthfulness.

Finally, we would like to highlight the strong association between the immune system gene sets and human skin youthfulness identified in this study. The immune system is known to change with age in humans [41], with manifestations ranging from reduced response to vaccinations [42–44] to changes in T cell diversity and function [45]. In the skin, clinical manifestations of immune aging may be observed through increased susceptibility to cutaneous infections and cutaneous malignancies [46]. In our gene expression analysis, the most significant gene sets pertained to immunological function, namely vaccine response, thymocyte development, and FOXP3 (Forkhead Box P3+, a master regulator in the development and function of regulatory T cells (Tregs) [47].

Tregs have been reported as increased in chronologically older human skin compared to younger skin [48,49]. The SY group in this study displayed an overall decrease in FOXP3 gene sets compared to non-SY group after adjustment for the effects of chronological age. It could be hypothesized that the SY group may have suppression of Tregs and increased skin immunity, a topic that requires future study.

Data emerging on blunted vaccine response in older individuals indicates that alterations in thymocyte development may play a role. For instance, a recent study involving immune response to yellow fever vaccine showed that elderly individuals displayed decreased CD4+ thymic emigrants and peripheral dendritic cells, leading to diminished responsiveness to vaccinations [50]. However, a direct connection between altered immune function and a phenotype like skin appearance remains to be explored. Recently, a genome wide association of skin youthfulness identified an association between SY and a single-nucleotide polymorphism adjacent to a voltage-gated potassium channel in skin dendritic cells [3]. Direct immunological functional studies on SY individuals versus non-SY individuals are needed to assess for differences in these two groups, to better understand whether and how functional differences lead to SY phenotype.

## Supporting Information

S1 Fig Determination of skin youthfulness phenotype. (A) Correlation between chronological age and delta (defined as chronological cage minus the skin age score). (B) Scatterplot of chronological age versus residual of delta. The top 10% of individuals with the largest residual of delta (red dots) were defined as SY individuals. This corresponded to SY individuals with residual of delta greater than or equal to 10.
(TIF)

S2 Fig Heat map and Gene Ontology analysis of significantly altered genes in chronological aging. (A) Heat map of gene expression profiles (n = 91) show significant changes (p<0.01 level) with chronological age, particularly starting at about age 40 years. Each column on the x-axis represents an individual, with chronological ages increasing to the right. The complete list of genes corresponding to the heat map is listed in S1 Table. (B) Gene ontology (GO) analysis of chronological aging genes. Biologic themes most significantly increased with age include cell and biological adhesion, positive regulation of immune response to stimulus, activation of immune response and extracellular matrix organization. The top nine biologic themes most significantly decreased with age involve genes related to pigmentation. The complete list of GO terms in order of p-value is shown in S2 Table.
(TIF)

S3 Fig The genes for SY and chronological aging are different, with only minimal overlap. (TIF)

S1 Table Gene transcripts altered with chronological aging. (A) List of gene transcripts increased with chronological age (n = 186). Transcripts are ordered by p<0.01, and ordered by p-value. (B) List of gene transcripts decreased with chronological age (n = 156) using p<0.01, and ordered by p-value. FC = fold change. P-values were adjusted for multiple hypotheses testing as indicated.
(XLSX)

S2 Table Gene Ontology terms altered with chronological aging. (A) Full list of Gene Ontology terms increased with chronological skin aging. (B) Full list of Gene Ontology terms decreased with chronological skin aging.
(XLSX)

S3 Table MSigDB collections used in our current study, as defined by Genomica. (XLSX)

S4 Table Gene transcripts altered with skin youthfulness. (A) Full list of SY genes, in order of significance. Genes that are increased with SY are shown in (B). Genes that are decreased with SY are shown in (C).
(XLSX)
