# Supplementary figures and images for: Novel Gene Expression Profile of Women with Intrinsic Skin Youthfulness by Whole Transcriptome Sequencing
Source: PLoS One. 2016 Nov 9;11(11):e0165913. doi: 10.1371/journal.pone.0165913 (PMC5102383; doi:10.1371/journal.pone.0165913)

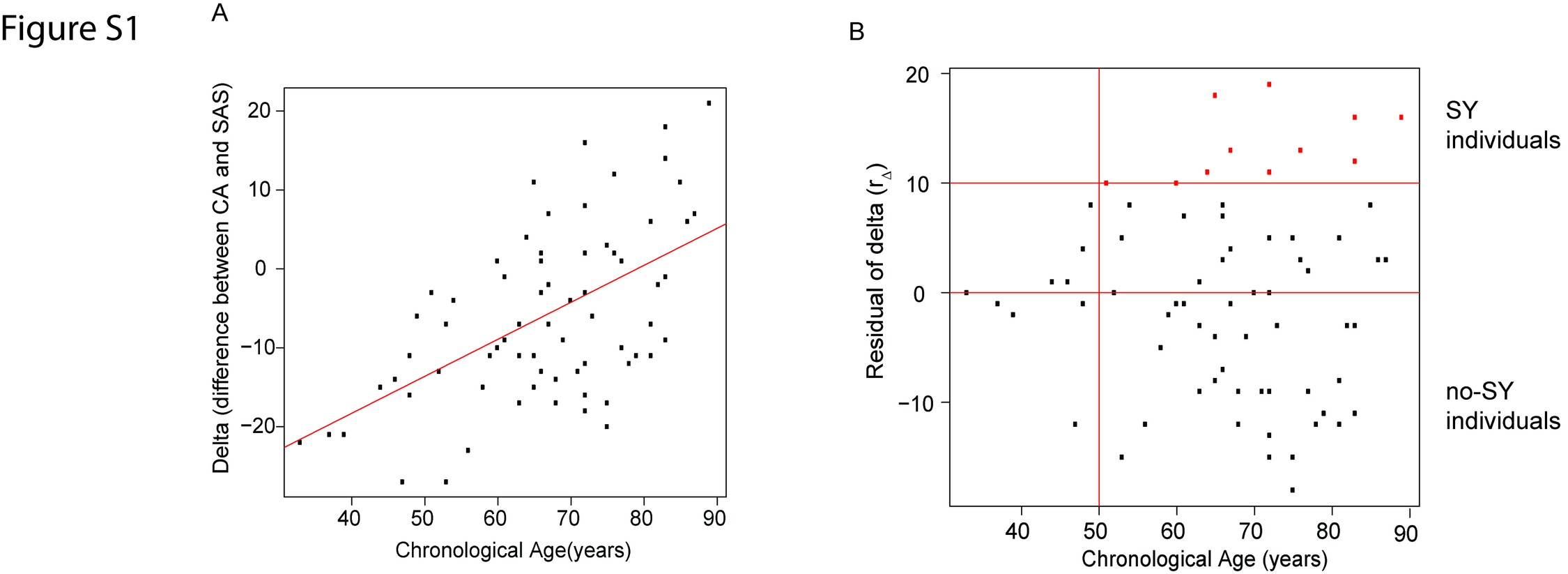

Supplement: S1 Fig — (A) Correlation between chronological age and delta (defined as chronological cage minus the skin age score). (B) Scatterplot of chronological age versus residual of delta. The top 10% of individuals with the largest residual of delta (red dots) were defined as SY individuals. This corresponded to SY individuals with residual of delta greater than or equal to 10. (TIF) [file pone.0165913.s001.tif]

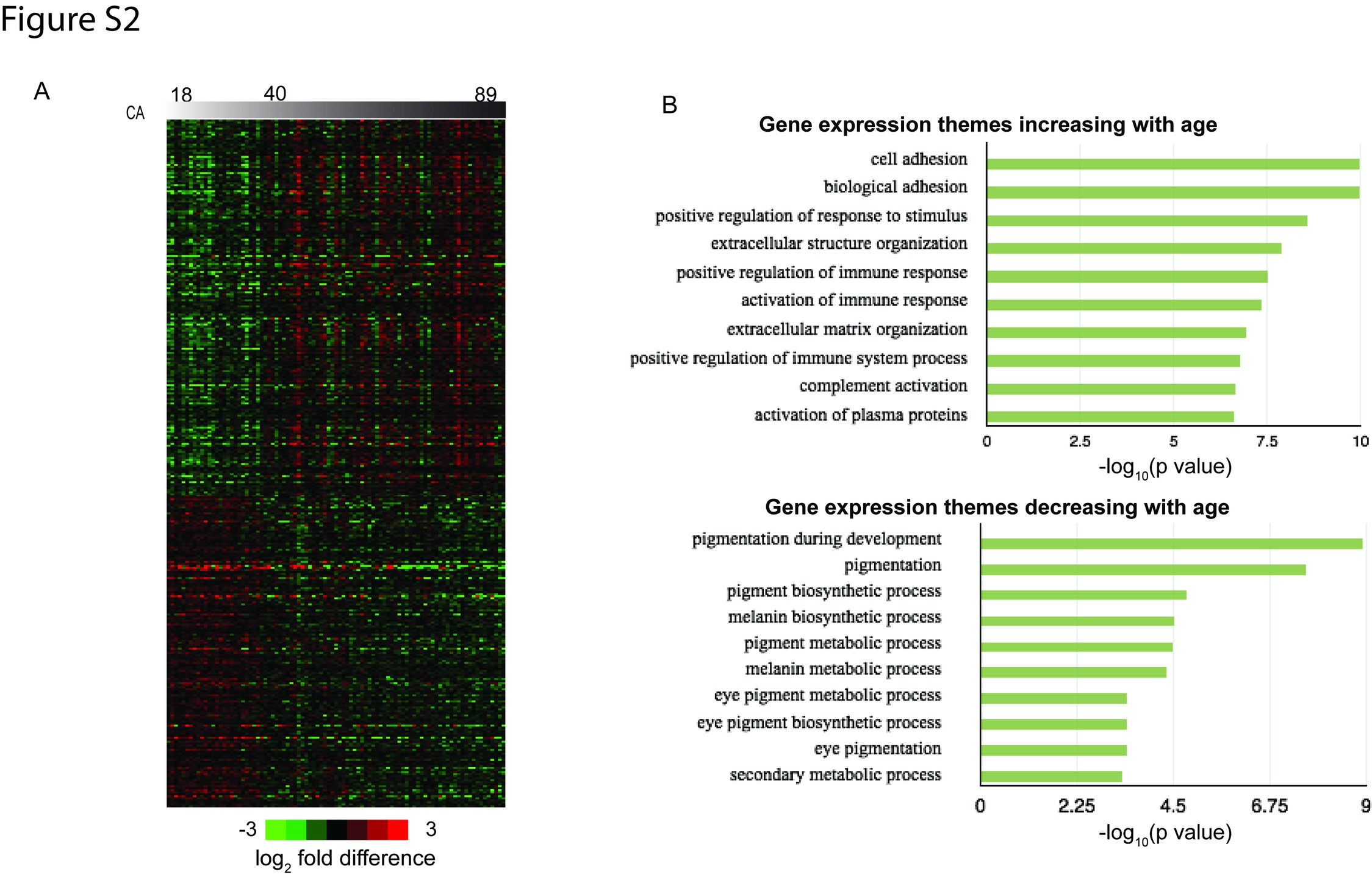

Supplement: S2 Fig — (A) Heat map of gene expression profiles (n = 91) show significant changes (p<0.01 level) with chronological age, particularly starting at about age 40 years. Each column on the x-axis represents an individual, with chronological ages increasing to the right. The complete list of genes corresponding to the heat map is listed in S1 Table. (B) Gene ontology (GO) analysis of chronological aging genes. Biologic themes most significantly increased with age include cell and biological adhesion, positive regulation of immune response to stimulus, activation of immune response and extracellular matrix organization. The top nine biologic themes most significantly decreased with age involve genes related to pigmentation. The complete list of GO terms in order of p-value is shown in S2 Table. (TIF) [file pone.0165913.s002.tif]

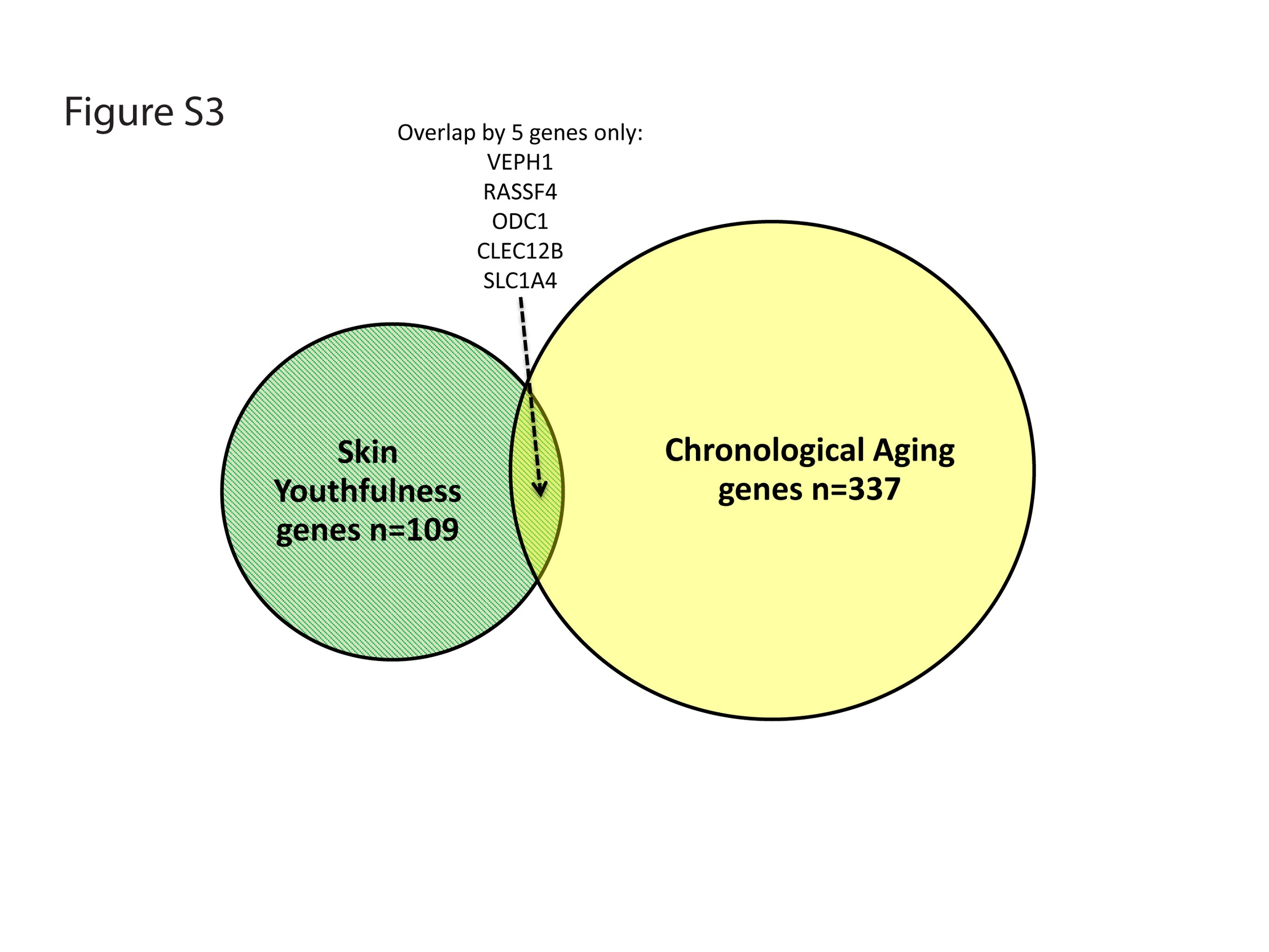

Supplement: S3 Fig — (TIF) [file pone.0165913.s003.tif]
